# Improving Prediction of Prostate Cancer Recurrence using Chemical Imaging

**DOI:** 10.1038/srep08758

**Published:** 2015-03-04

**Authors:** Jin Tae Kwak, André Kajdacsy-Balla, Virgilia Macias, Michael Walsh, Saurabh Sinha, Rohit Bhargava

**Affiliations:** 1 Center for Interventional Oncology, National Institutes of Health, Bethesda, MD 20892, USA; 2 Department of Computer Science, University of Illinois at Urbana-Champaign, Urbana, IL 61801, USA; 3 Beckman Institute for Advanced Science and Technology, University of Illinois at Urbana-Champaign, Urbana, IL 61801, USA; 4 Department of Pathology, University of Illinois at Chicago, Chicago, IL 60612, USA; 5 Department of Bioengineering, Mechanical Science and Engineering, Electrical and Computer Engineering, Chemical and Biomolecular Engineering and University of Illinois Cancer Center, University of Illinois at Urbana-Champaign, Urbana, IL 61801, USA

## Abstract

Precise Outcome prediction is crucial to providing optimal cancer care across the spectrum of solid cancers. Clinically-useful tools to predict risk of adverse events (metastases, recurrence), however, remain deficient. Here, we report an approach to predict the risk of prostate cancer recurrence, at the time of initial diagnosis, using a combination of emerging chemical imaging, a diagnostic protocol that focuses simultaneously on the tumor and its microenvironment, and data analysis of frequent patterns in molecular expression. Fourier transform infrared (FT-IR) spectroscopic imaging was employed to record the structure and molecular content from tumors prostatectomy. We analyzed data from a patient cohort that is mid-grade dominant – which is the largest cohort of patients in the modern era and in whom prognostic methods are largely ineffective. Our approach outperforms the two widely used tools, Kattan nomogram and CAPRA-S score in a head-to-head comparison for predicting risk of recurrence. Importantly, the approach provides a histologic basis to the prediction that identifies chemical and morphologic features in the tumor microenvironment that is independent of conventional clinical information, opening the door to similar advances in other solid tumors.

Prostate cancer is the second leading cause of deaths following lung cancer in US men and constitutes one-third of non-skin cancer diagnoses every year^1^. One of the most acute needs in PCa management today is higher precision in prediction of clinical outcomes for more effective decision-making^2^. Various prediction tools have been developed for this purpose and they largely rely on patients' clinical, pathologic and demographic data^3^. These data include age, PSA level, Gleason grade, and pathologic stage. The available tools include risk grouping^4^, look-up tables^5^, machine learning^6^, nomograms^7^,^8^, and risk scoring^9^,^10^. The performance of these predictive tools is more consistent and superior to manual decisions; hence, they are implemented in modern patient care. Currently, the widely-validated Kattan nomogram^7^,^8^ and CAPRA-S score^10^ are considered as the best performing tools for prostate outcome prediction after radical prostatectomy (RP). The accuracy of prediction is often measured using a receiver operating curve (ROC) and the area under the curve (AUC) to segment patients with poor and good outcomes. The modest overall accuracy of available tools is further devalued by the realization that the tools largely fail for the most common cohort in the modern era (mid-grade, confined disease with moderate PSA level) and there is a tendency to overestimate likelihood of disease recurrence for lower risk patients^10^,^11^. Incorporating additional clinical variables has not significantly improved predictions^12^. Likewise, integrating gene expression^13^,^14^, immunohistochemistry^15^,^16^, magnetic resonance imaging^17^,^18^,^19^ or tissue morphology^20^,^21^ data has met with limited success^2^ and none has achieved widespread clinical acceptance.

While most of the diagnostic and prognostic efforts have focused on epithelial cells, an important role for the tumor microenvironment or stroma in cancer progression has also been reported^15^,^20^,^21^,^22^. No methods are available, however, to make anecdotal and laboratory observations about the microenvironment into a practical clinical test. The challenges of measuring multiple cell types, via multiple, cumbersome steps, and associating the data manually from multiple sections and with clinical management has limited the potential of this promising avenue. Here, our first aim was to establish an imaging approach that promises to change this deficiency. We use chemical imaging, which is strongly emerging as a platform for combining spatial and molecular analysis. Fourier transform infrared (FT-IR) spectroscopic imaging^23^, in particular, combines the spatial specificity of optical microscopy with the molecular selectivity of vibrational spectroscopy. The IR absorption spectrum at each position (or pixel) is a quantitative record of chemical composition^24^ and FT-IR imaging has been applied to study various cell types and different types of cancers^25^,^26^,^27^,^28^,^29^,^30^, including that in the prostate^31^,^32^,^33^. In particular, chemical changes in both epithelial cells^34^ and cancer-associated stroma^35^,^36^ were recently reported using FT-IR spectroscopic imaging^37^. While promise in imaging different cell types has been shown, the use of IR imaging for prognosis has not been previously proposed. In particular, the question of prostate cancer recurrence has not been addressed, which is the second aim of this study. We hypothesized that the chemical properties of the tissue, and especially of the stroma, may add additional information that can improve prediction. To test this hypothesis in the most pressing area of contemporary PCa need, we employed a recurrence-enriched and mid-grade dominant cohort^38^ where recurrent cases and non-recurrent controls were matched for age at prostatectomy, race, Gleason score, and pathologic (pTNM) stage. This cohort not only provides the most challenging and demanding subset for modern prostate pathology but also facilitates discovering novel predictors of cancer recurrence which are independent of the conventional clinical parameters (age, race, Gleason score, and pathologic stage). This is an exceptionally challenging cohort that requires us to also change analytical methods, which was the third aim of our study. Biological variation has been shown to be a primary source of variation in a tissue^39^, which is exacerbated with heterogeneity in cancer^16^. Hence, conventional approaches in analyzing chemical imaging data^27^,^28^,^40^ are inappropriate for this application. Therefore, we propose to use a new approach called “frequent pattern mining”^41^ to discovers subsets, subsequences, or substructures in a dataset^42^ that are not apparent using simple dimensionality reduction. To make the final diagnosis or predict risk score, we adopt a Ranking-Support Vector Machine^43^ such that a recurrent case is ranked at higher risk than a non-recurrent control. Finally, the fourth part of our study compares the new approach to the current gold standards - Kattan nomogram and CAPRA-S score – to determine if the approach could yield improved PCa outcome predictions. It is notable that the design of the study addresses a pressing need, utilizes new ideas to examine cancer by focusing on both the tumor and microenvironment and our approach is entirely compatible with other tests – whether MRI-based non-invasive assays or digital pathology^44^,^45^ on the same tissue specimens.

## Results

### Stromal IR features distinguish recurrence cases from non-recurrence controls

**Figure 1:**
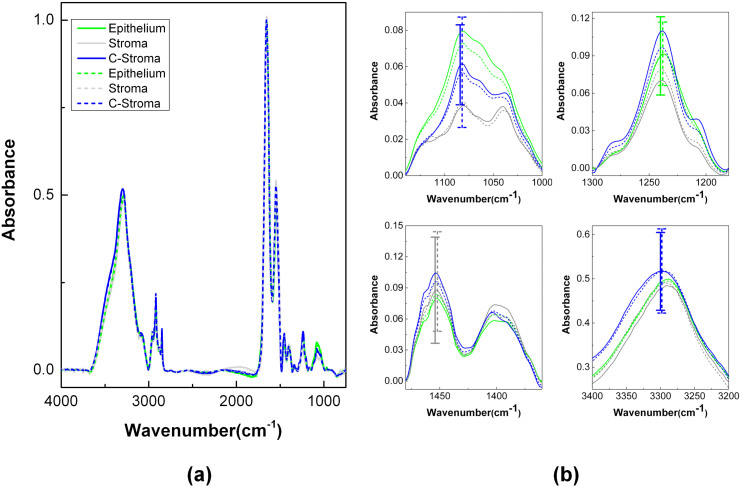
IR Spectra. (a) IR spectra (4,000–720 cm^−1^) of epithelium (green), distal stroma (grey; Stroma) and adjacent stroma (blue; C-Stroma) to tumor. (b) A close-up view of four different spectral areas (1,140–1,000 cm^−1^, 1,300–1,180 cm^−1^, 1, 480–1,360 cm^−1^, and 3,400–3,200 cm^−1^) and error bars at the spectral peaks. Solid and dotted line represent recurrence case and non-recurrence control, respectively.

**Figure 2:**
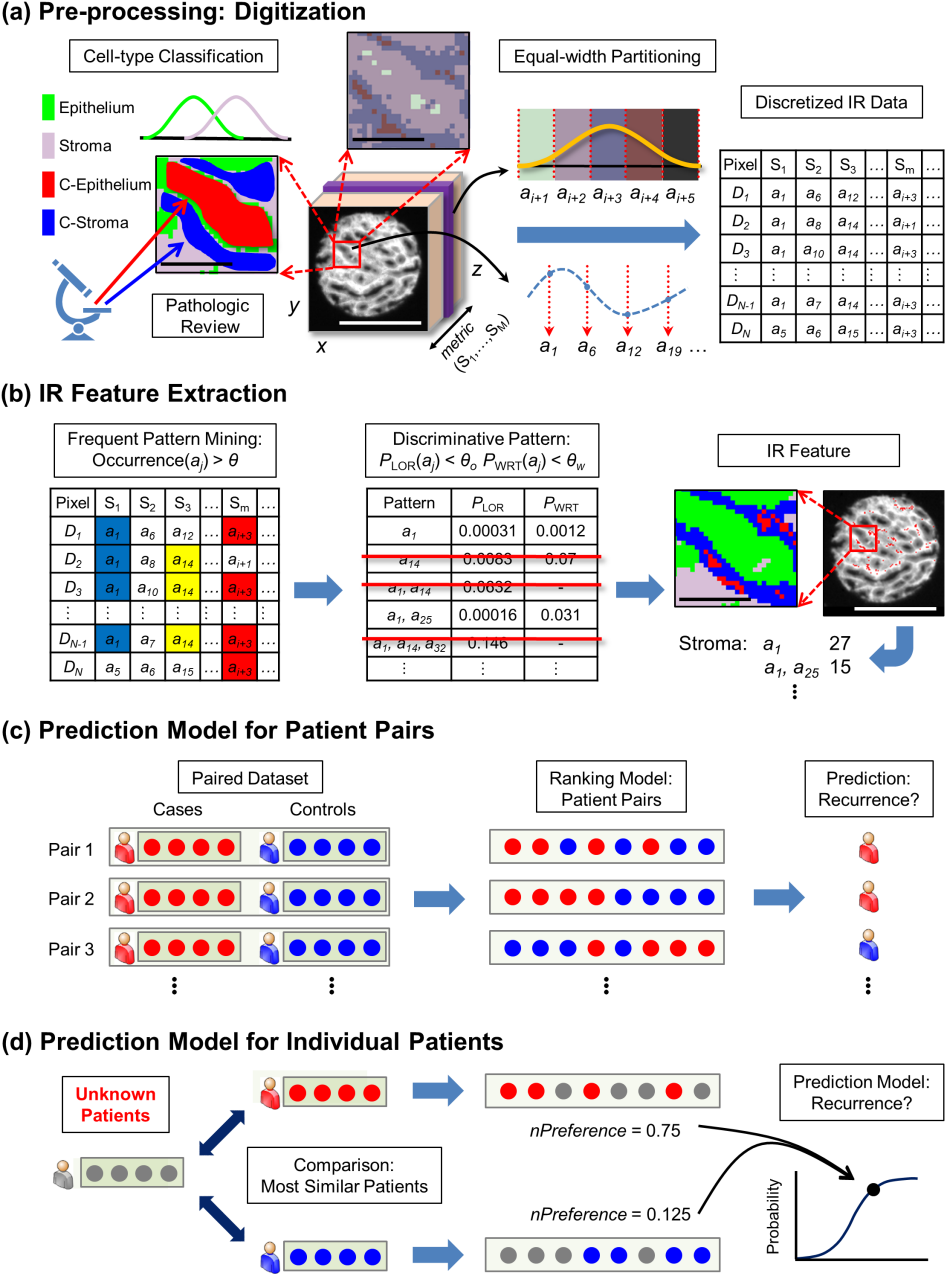
Overview of Outcome Prediction. Given an IR image, (a) cell-types (Epithelium and Stroma) are identified by IR imaging and, by pathologic review, cancer epithelial cells (red; C-Epithelium) and adjacent stromal cells (blue; C-Stroma) are manually selected and discretized. Upon discretization, pixels in tissue contain a set of discrete values representative of the bins. (b) Applying frequent pattern mining and statistical tests, IR features are extracted. Using the features, prediction models for (c) patient pairs and (d) an individual patient are formed and predictions are made. C-Epithelium and C-Stroma denote the selected cancerous epithelium and stroma next to it, respectively. *nPreference* is a normalized preference score for the unknown patient against the most similar patient. Black and white bars denote 100μm and 500μm, respectively.

Five tissue microarrays (TMAs), including 186 patients, were imaged to provide patient and clinical diversity for the most difficult contemporary cohort of patients (predominantly mid-grade and organ confined disease). The characteristics of the patient set are detailed in Table 1 and data acquisition and analysis protocols are detailed in the methods section. We first classified the tissue into different cell types following previous protocols^32^ and compared the average IR absorption spectra (>500 pixels) of epithelium and two types of stroma (distal and adjacent to tumor) to discern differences between recurrent cases and non-recurrent controls. There was no significant difference between recurrent cases and non-recurrent controls for different cell types (Figure 1). Transitioning from average values to examining each patient, two separate sets of analyses were conducted – on epithelial cells and on stromal cells. Based on both population difference and individual variation, IR spectra were converted into 98 spectral metrics that are individually indicative of molecular content in tissue (Supplementary Table S1). The spectral features from each cell type were separately handled in our data analysis pipeline (Figure 2) since cell types greatly differ in morphology, chemistry, and function. From data for each cell type, we built a machine learning classifier that utilizes not only an individual IR metric but also the combination of IR metrics, designated as “patterns” (Supplementary Figure S1). The present approach to machine learning is to simply treat patterns from all pixels of a sample as equal – as assumption that does not hold if there are sub-classes of pixels, outliers, or contaminated pixels. We recognize that a pattern could also identify a subset of pixels that may share (un)known biochemical functions or represent sub-cellular components within a sample. The subset of pixels, we hypothesize, has predictive power that may be lost in examining average values from all cells, even of the same type. The use of patterns can make the analysis more sensitive by not focusing on small differences in absorption that may arise from natural variation or sampling and make the analysis more robust by focusing on biochemical characteristics or metric expression. The frequency of a pattern, i.e., the occurrence of a subset of pixels, is used as a feature in our classification algorithm. Numerous patterns can be generated, but we do not know *a priori* which patterns may represent the important biochemical functions or sub-cellular components. We adopted a pattern selection step^46^ that automatically identifies the useful patterns from training data, before using such patterns in constructing the final classifier. The classification algorithm used in this study is a Ranking-Support Vector Machine (SVM)^43^. Details of the classification scheme are provided in the Methods section.

To ensure robustness, we tested the classifier in a variety of ways: by cross-validation on a calibration data set as well as by training on the calibration data set and testing on a separate validation dataset. The former uses a nested case-control study design, where a recurrence case is matched with a non-recurrence control, and the latter adopts a non-nested design. For both validations, the testing dataset and outcome were blinded to the classification model. We also note that multiple TMA slides are used, which includes variance from sample preparation and handling, with a blinded selection of patients into a calibration and a validation set. We detail these multiple modes of evaluation next. We first tested the classifier by *K*-fold cross-validation (*K* = 10) on the calibration dataset (120 patients, see Methods), using a nested design. In this test, the classifier was always presented with a case-control pair of patients that were matched by clinical descriptors (age at prostatectomy, race, Gleason score, and pathologic stage), and was asked to discriminate the case from the control. As is standard in cross-validation, the dataset was divided into *K* equal-sized partitions and one partition was left out as the “test partition”. The classifier was trained on the union of the remaining *K-*1 partitions and made to predict outcomes in the test partition. After repeating this *K* times with systematically different choices of the left-out partition, correct and incorrect predictions were summarized. The classifier based on epithelial data was not capable of distinguishing recurrent cases from non-recurrent controls (~50% accuracy). On the other hand, the classifier based on stromal data discriminated cases from controls with ~70% accuracy (*P* < 0.001, Binomial test). Note that existing methods that rely on clinical variables to predict outcome are expected to perform close to a random classifier (50% accuracy) in this nested design sample set, since each case-control pair presented to the classifier was identical/matched in terms of many clinical variables.

We next performed evaluations on the same data set as above but using a non-nested design, where the goal is to predict outcome of unknown individual patients (query). This was done with a modification of the classifier discussed above. For any test query, the algorithm finds the most similar recurrent and non-recurrent patients from the training dataset using clinical variables alone, and compares them to the query as detailed in Methods. The evaluations are done with *K-*fold cross validation as above, and in each “fold” the *K-1* partitions other than the left out partition serve as training data. The predicted probability of recurrence (PPR) of the test patient based on these comparisons is computed using a logistic regression model. We call this PPR value the “IR Score” of the patient, as described in Methods. The sensitivity and specificity of the classifier are summarized using a receiver operating characteristic (ROC) curves and by the area under the ROC curve (AUC). We also calculate a 95% confidence interval (CI) by Bootstrap re-sampling for comparison. The classifier using data only from epithelial pixels could not appreciably distinguish the query as recurrent or non-recurrent (0.60 AUC, 95% CI: 0.50–0.69), but the classifier based on stromal data was effective with an AUC of 0.71 (95% CI: 0.61–0.81) in the calibration dataset (120 patients) in *K*-fold cross-validation (*K* = 10). After evaluating predictive performance on the calibration data set of 120 patients, we subjected the classifier to a separate, completely independent validation dataset of 66 patients. The IR Score of each patient in the validation dataset was recorded and an AUC of 0.57 (95% CI: 0.43–0.72) was obtained when using epithelium pixels. Using stromal pixels, an AUC of 0.71 (95% CI: 0.57–0.83) was observed, indicating that the test is robust.

### IR Score outperforms popular existing tools in predicting outcomes

**Figure 3:**
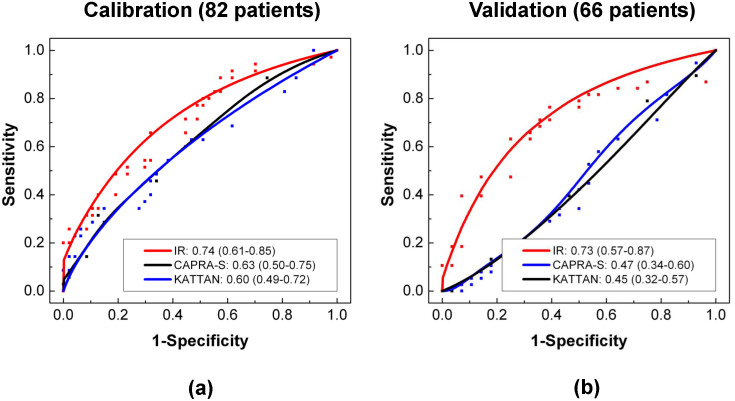
Performance of Outcome Prediction. Outcome prediction of three models – CAPRA-S (black), Kattan nomogram (blue), and IR (red) – on (a) the calibration dataset and (b) the validation dataset. AUCs and 95% confidence intervals in parentheses are shown on the plots. Lines represent the smoothed ROC curves and rectangular dots denote the empirical sensitivity and 1-specificity points.

We compared the performance of the IR Score to the two of the most commonly used clinical tools (Kattan nomogram and CAPRA-S score) for predicting outcomes after radical prostatectomy. Here, 82 patients, who have no record of neoadjuvant or adjuvant therapy, from the calibration dataset were employed since Kattan and CAPRA-S score are only applicable to such patients. As shown in Figure 3a, the IR Score (AUC = 0.74, 95% CI: 0.61–0.85) outperforms both Kattan (AUC = 0.60, 95% CI: 0.49–0.72) and CAPRA-S (AUC = 0.63, 95% CI: 0.50–0.75) scores when evaluated on the calibration dataset (82 patients). In a similar evaluation on the independent validation dataset (66 patients), Kattan (AUC = 0.45, 95% CI: 0.32–0.57) and CAPRA-S (AUC = 0.47, 95% CI: 0.34–0.60) scores performed far worse than the IR Score (AUC = 0.73, 95% CI: 0.57–0.87) as seen in Figure 3b. We note that these comparisons were from the individual query method only, since the nested case-control design is not amenable to better-than-random classification by Kattan or CAPRA-S scores, by definition.

**Figure 4:**
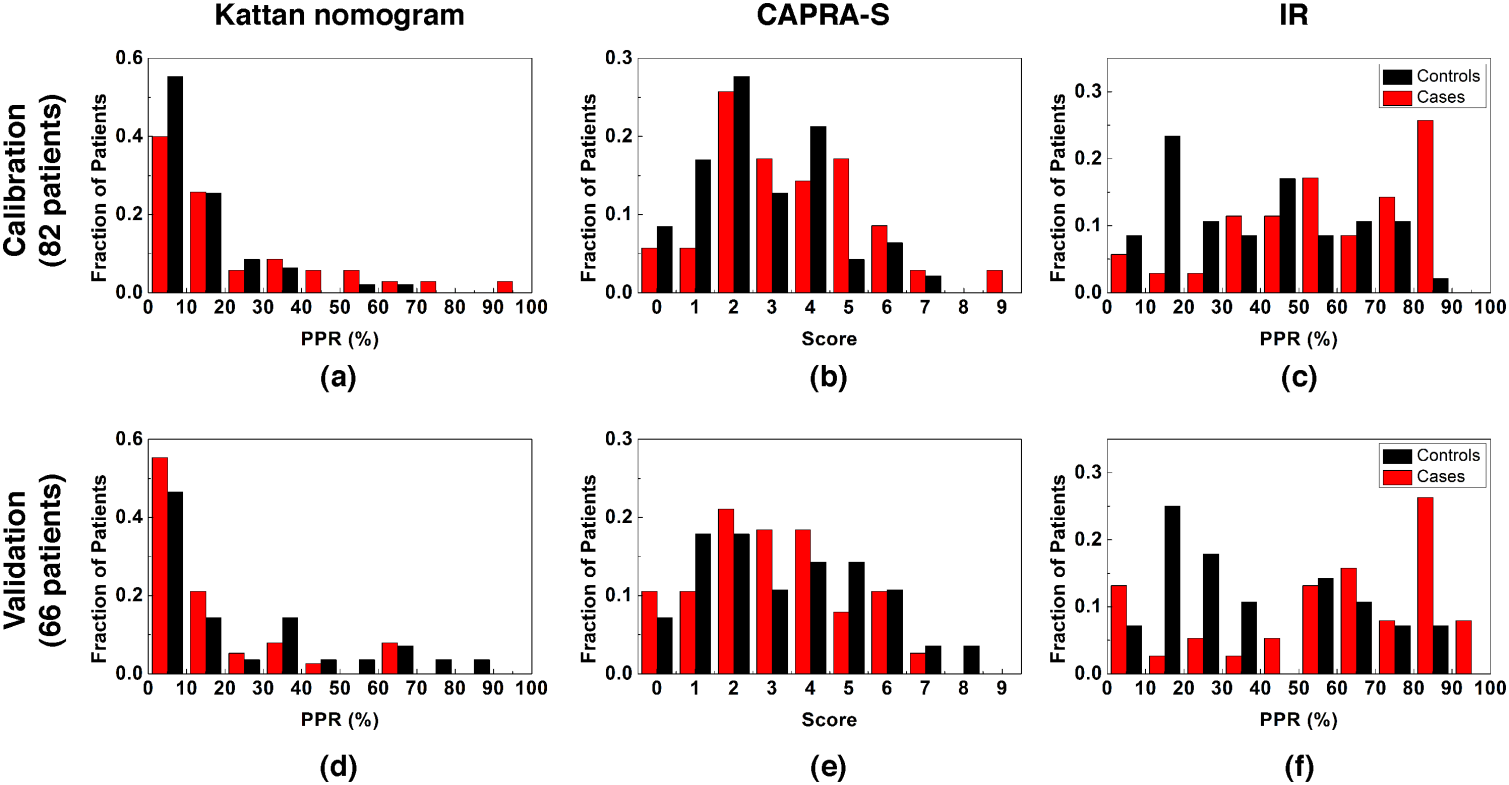
Score distribution. Distribution of the recurrence cases (red) and non-recurrence controls (black) by (a)(d) Kattan (b)(e) CAPRA-S (c)(f) IR Scores. Distributions on (a)(b)(c) the calibration dataset and (d)(e)(f) the validation dataset are plotted. PPR represents the predicted probability of recurrence.

We next compared the distribution of the patients according to the IR Score used by our algorithm for predicting recurrence. We compared the IR Score distributions of cases and controls to analogous distributions from both the Kattan nomogram and CAPRA-S score (Figure 4). PPR values represented by the IR Score had distinct distributions for recurrence cases versus non-recurrence controls (*P* ≈ 0.02, Kolmogorov-Smirnov test). In contrast, the distributions of either the Kattan or the CAPRA-S scores were indistinguishable between the two classes (*P* > 0.1, Kolmogorov-Smirnov test). A majority of the patients, regardless of recurrence status, had very low PPR values using the Kattan nomogram. Most samples were assigned to either low-risk (score 0–2) or intermediate-risk (score 3–5) category by CAPRA-S score. This shows that the discriminative capability of the two widely-used tools in this challenging data set is very limited, and demonstrates the potential of the new approach proposed here to add to clinical decision-making.

### Stromal IR features are independent predictors of cancer recurrence

The relationship between IR Score and recurrence was investigated in the context of conventional clinical variables (age at prostatectomy, Gleason grade, pathologic stage, and PSA level). In particular, we asked if the IR Score and clinical variables together can better predict recurrence than clinical variables alone can. If this is the case, then there must be additional information in the IR Score that is not present in clinical variables. By fitting a logistic regression model of recurrence using IR Score and the clinical variables as covariates, we estimated the strength of the association between IR Score and cancer recurrence, regardless of the clinical variables, on the calibration dataset (see Supplementary Information for details). IR Score was used as either a continuous or a categorical variable. As a continuous variable, the strength of the association with cancer recurrence was examined for a one-unit increase in IR Score as fixing the clinical variables. As a categorical variable, patients were assigned to quartiles (1–4) by IR Score and, fixing the clinical variables, the relationship with cancer recurrence was compared between the patients in the lowest quartile and the patients in other quartiles (Table 2). For both types, a significant association was observed (*P* < 0.001 as a continuous variable and ~21-fold differences between the lowest quartile and the highest quartile as a categorical variable). These indicate that higher IR Scores are significantly correlated with higher likelihood of recurrence, in ways that are not captured by clinical variables, i.e., independent from these variables. The association between Kattan and CAPRA-S scores with cancer recurrence was not significant (*P* > 0.06 as a continuous variable and ~2- to 6-fold differences between the extreme quartiles as a categorical variable; Wald chi-square test) when tested.

### IR Score adds independent predictive power to popular existing tools

As shown above, the relationship between IR Score and cancer recurrence is independent of clinical variables that the existing tools rely upon. To further test this hypothesis, we combined IR Score with Kattan and CAPRA-S scores as follows:
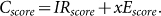
*C_score_* and *IR_score_* denote combined score and IR Score respectively, while *E_score_* is either Kattan score or CAPRA-S score and *x* is a weight for the scores (0 < *x* ≤ 1). As changing a weight *x* from 0 to 1, the performance of the combined score *C_score_* was measured by computing AUC. Increasing *x*, the performance of the combined score only marginally improved upon the performance of IR Score alone (even degraded capability in the combination of IR Score and Kattan score on the validation dataset) and the improvement was not statistically significant (Supplementary Figure S2). Combined with Kattan and CAPRA-S scores, the AUC of IR Score reached 0.75 (95% CI: 0.64–0.85) and 0.75 (95% CI: 0.63–0.85) respectively on the calibration dataset, whereas IR Score alone achieved AUC of 0.74 (95% CI: 0.61–0.85). Moreover, on the validation dataset the combined score led to degradation in classification performance. An AUC of 0.65 (95% CI: 0.50–0.79) and 0.74 (95% CI: 0.58–0.87) was attained by combining IR with Kattan and CAPRA-S scores respectively, while an AUC of 0.73 (95% CI: 0.57–0.87) was achieved by IR Score alone. Hence, the IR Score is truly independent of the existing tools.

### IR features correspond to specific functions in stroma

**Figure 5:**
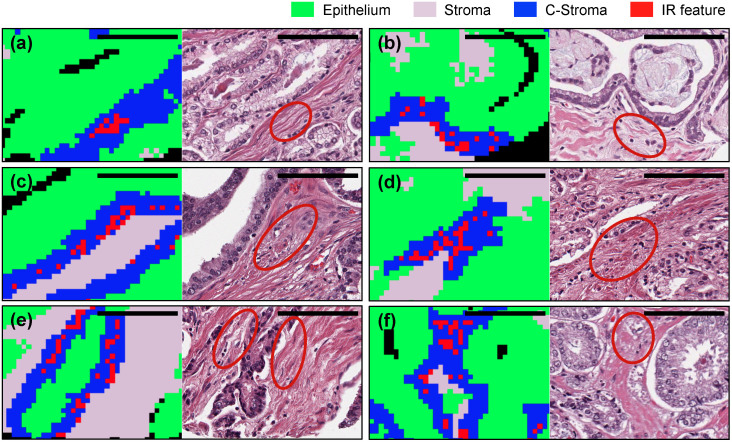
Comparison of IR and H&E images. IR stromal features (red rectangles) and their corresponding areas (red circles) are marked on IR and H&E images, respectively. (a)(c)(e) Recurrence images and (b)(d)(f) non-recurrence images. C-Stroma represents stroma cells adjacent to cancer cells. Black scale bar denotes 100μm. IR features are related to (a)(b)(e)(f) loose or myxoid stroma, (b)(c)(d)(e)(f) fibroblasts, and (a)(d)(e)(f) retraction clefting.

We examined the data to understand the underlying spectral and spatial basis of our successful classification. Examining the metrics, two prominent spectral regions were recognized. The peaks in 3000–3600 cm^−1^ range (Figure 1b) were present in all of the features; these are related to N-H stretching vibrational modes (in proteins) or O-H stretching vibration^47^,^48^. About half of the features were related to the peaks in 990–1132 cm^−1^, which include C-O stretching vibrational mode at 1042 cm^−1^ due to oligosaccharides^49^. The complete list of IR metrics useful in outcome prediction is available in Supplementary Table S1, indicated by a “Yes” in whether the feature was found to be useful. While the chemical content is elucidated, the spatial origin of our differences is also enlightening. Spectra can be used to find regions of the image most closely associated with prediction. Pixels containing the discriminating stromal features are identified and compared to their corresponding H&E images (Figure 5). The features appear in the regions where reactive stroma is present. More specifically, loose or myxoid stroma and fibroblasts are often observed around the pixels that were most useful in outcome prediction. Stromal reaction (or desmoplasia), which occurs in most solid human cancers^50^, contains increased number of fibroblast and myofibroblasts and modifies extracellular matrix composition. We also found that the discriminating features are frequently present next to periacinar retraction (clefting), a separation of the gland from the adjacent stroma that has been associated with cancer recurrence^51^.

## Discussion

We have presented a novel approach for precise predictions in prostate cancer using a combination of emerging IR imaging and a novel computational approach. Stromal features, not epithelial features, were found to be more significant in distinguishing biochemical recurrence cases from non-recurrence controls for both nested and non-nested case-control designs. While these results indicate that the stroma contains molecular signatures related to cancer progression, as has been shown in the past; here, however, we have not used any dyes, molecular stains or human interpretation. Automated methods that can comprehensively analyze the entire tumor microenvironment lead to a robust and accurate predictive model. This presents a new opportunity for combined molecular and imaging-based outcome predictions, as opposed to simply using clinical data or molecular biomarker techniques^52^. In a head-to-head comparison, IR-based prediction outperforms both Kattan nomogram and CAPRA-S score on multiple datasets. IR prediction is especially exciting as the patient cohort here is of clinically difficult cases, where nomograms are of limited utility. There were slight differences in the performance of Kattan and CAPRA-S scores for the calibration dataset and the validation dataset. This may be attributable to the differences in clinical characteristics of the patients (Table 1). For instance, in the validation dataset, differences in age and PSA level between recurrence cases and non-recurrence controls are smaller compared to those in the calibration dataset. More recurrence cases than non-recurrence controls (17 recurrence cases and 6 non-recurrence controls) with lower Gleason sum (≤6) exist in the validation dataset. All of the 17 recurrence cases were designated as either lower-risk (70%) or intermediate-risk (30%) category by CAPRA-S score. The recurrence cases had relatively lower PPR values (88% had <10% PPR) by Kattan nomogram. However, the performance of IR Score was consistent and the separation was evident by IR Score; at the level of PPR ≤30%, ~3-fold more non-recurrence controls were observed than recurrence cases; 2- to 4-fold more recurrence cases were found at ≥70% PPR. Overall, the patients, grouped into an intermediate (or lower) risk group by the conventional tools, can be further stratified by IR Score whereas both Kattan and CAPRA-S scores were not contributory. Since Kattan and CAPRA-S scores did not perform well, it is not surprising that the combined score with IR Score was not able to significantly improve upon IR Score alone. The synergy between IR Score and the two scores does not lie in a simple combination. We believe that the way to utilize IR Score is to add it to the existing tools as an additional factor and to improve the utility of the tools. This is especially true for the clinically difficult intermediate risk where Kattan and CAPRA-S methods fail, as demonstrated by the cohort here. The method presented is likely to prove an exciting addition to the power of Kattan and CAPRA-S methods and could substantially increase their predictive power.

In addition to Kattan and CAPRA-S scores, several imaging- and molecular-based techniques have been developed; however, the relationship between IR Score and these techniques is yet unclear. This is largely due to the non-availability yet of the data and/or images on the same set. The approach presented here is entirely compatible with these approaches, including pre-operative MRI-based approaches^53^, downstream genomic tests^54^, emerging mass spectrometric^55^,^56^ or even other intra-operative^57^ or emerging vibrational spectroscopic^58^ approaches. None of these techniques offers the unique microenvironment profiling and imaging capability of IR microcopy, using protein, nucleic acid and carbohydrate signals inherent in the spectroscopic capability used here. The presented data are both unique and complementary; hence, it will be desirable to examine if there is a synergy between IR Score and predictive features from other techniques. Finally, both the chemical and biological underpinnings of the discriminative stromal spectroscopic features uncovered by the approach here provide further avenues for examination. The features may be related to the molecules pertaining to (myo)fibroblasts, extracellular matrix composition or retraction clefting; for instance, chondroitin sulfate, known to be associated with prostate cancer progression^59^. However, the specific molecules or chemical/biological causes relevant to the IR features cannot be identified without further biochemical assays such as immunohistochemistry, proteomics, or glycomics. In an emerging trend, obtaining higher spatial resolution IR imaging^60^ could improve the recognition of the features yielding a finer classification model and, in turn, achieve better predictions of recurrence. In summary, the presented approach not only shows tremendous promise to directly address a major contemporary need in PCa management but can also help narrow the focus for further research studies. The same approach may be used to examine other questions of importance in contemporary cancer care, for example, the determination of the cohort in which adjuvant therapy may prove useful, for examining the metastatic potential of organ-confined disease and for the analysis of solid tumors in other organs.

## Methods

### Samples and data preparation

This study and protocols were conducted as approved by the University of Illinois Institutional Review Board (IRB) via protocol #06684. Informed consent was obtained from all subjects included in this study. We used a set of five tissue microarray slides (TMAs) constructed by the NIH-sponsored Cooperative Prostate Cancer Tissue Resource (CPCTR)^38^. The TMA contain up to four cores each from 404 patients. Half the patients experienced biochemical recurrence after radical prostatectomy (cases) and the other half did not for at least 5 years after radical prostatectomy (controls). Cases and controls were matched for age at prostatectomy, race, Gleason score, and pathologic (pTNM) stage. ~90% of the patients are in the intermediate risk group using D'Amico risk grouping^4^, and 268 of the 404 patients have Gleason sum 7 – the most problematic and prevalent score. These TMAs, hence, are recurrence-enriched as well as mid-grade dominant, which are unique and challenging compared to other studies^7^,^8^,^9^,^10^ – but represent the most crucial subset where prediction capabilities are sorely desired. Further description of this outcomes-based TMA is available in Ref. 61.

For accurate diagnosis, tissues with insufficient malignant epithelial cells (<10% of core) were omitted by combined pathologic review and IR-based cell-type classification^32^. Stromal cells in the vicinity of epithelial cells (within ~50 μm) were also selected as we have previously noted using engineered tissue models^35^ that stromal reaction can be observed within a safe margin of 100 μm from the tumor. Finally, the study only included subjects who had at least two cores with both sufficient epithelial cancer cells and adjacent stromal cells. Hence, calibration dataset consists of 60 patient pairs, with a total of 380,685 and 333,167 epithelial pixels for the cases and controls, respectively (the “IR epithelium dataset”). Similarly, 101,576 and 95,925 stromal recurrence and non-recurrence pixels, respectively (the “IR stroma dataset”), were collected. For comparison with Kattan nomograms and CAPRA-S score, 82 patients (35 recurrence cases and 47 non-recurrence controls) who have no record of neoadjuvant or adjuvant therapy, were selected from the calibration dataset. Similarly, a completely independent “validation dataset” of 66 patients (38 recurrence cases and 28 non-recurrence controls) with 193,620 epithelial recurrence, 133,126 epithelial non-recurrence, 50,745 stromal recurrence, and 41,130 stromal non-recurrence pixels, respectively, was obtained. Clinical characteristics of the patient cohort are available in Table 1.

IR imaging data were acquired at a spatial pixel size of 6.25μm × 6.25μm, a spectral resolution of 4 cm^−1^ at an undersampling ratio of 2 using a Perkin-Elmer Spotlight imaging system. Each pixel's interferogram was processed using NB-medium apodization and spectra truncated to 4,000–720 cm^−1^. Tissue samples were microtomed to a thickness of ~5 μm. One section was placed on IR transparent BaF_2_ slides, while consecutive sections were placed on a standard glass slide and stained with hematoxylin and eosin (H&E) for pathologic review. H&E stained images were acquired on an optical microscope at 40× magnification. Since IR spectra are high-dimensional, we convert data into 98 spectral metrics (Supplementary Table S2). Metrics are defined by spectroscopic review and can be absorbance at a peak position, ratio between two peaks, and area and center of gravity of a spectral region^32^. Conversion to metrics not only reduces the dimensionality of the original data to a manageable size but also preserves the intrinsic and essential characteristics of the data while allowing interpretation of the results.

### Chemical feature extraction

Due to biological variation and heterogeneity in tissue and caner, signatures of cancer recurrence may not be apparent. Here, we hypothesize that the signatures of recurrence reside in a part of cells or tissues which share certain biochemical properties and can be identified by IR spectra. Using the combinations of discretized IR data, we attempt to find such signatures efficiently and effectively. Ranking approach is adopted to achieve an accurate and robust prediction of recurrence.

### Discretization

Each IR metric is independently discretized by dividing the entire range of that metric into a number of equal-sized partitions or “bins” (Figure 2a), and a representative value is designated for each bin. The value of the IR metric for a pixel is then transformed to the appropriate discrete value representative of the bin containing the original value. Thus, each pixel *d_i_* in a dataset *D* = {*d*_1_,*d*_2_,...,*d_n_*} (*n* pixels), originally a vector of IR metric values (*k* IR metrics), is now represented as a vector 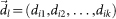 where each dimension *d_ij_* is the discretized value of a distinct IR metric *j* corresponding to pixel *d_i_*. We used 20 bins to discretize each IR metric. Transformation of a continuous IR metric into a discrete format further bounds the joint probability distributions of IR metrics and helps mitigate the effect of variance by discarding the unnecessary precision.

### Frequent pattern mining

A “pattern” is a specification of values for a certain subset of dimensions, e.g., a pattern ϕ = ((2, ϕ_2_), (5, ϕ_5_), (6, ϕ_6_)) specifies that the second dimension has value ϕ_2_, the fifth dimension has value ϕ_5_, etc, and a vector 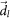 is said to match this pattern if *d_i2_ = * ϕ_2_, *d_i5_ = * ϕ_5_, and *d_i6_ = * ϕ_6_. A frequent pattern is a pattern that is matched by many pixels (Figure 2b), i.e., the number of pixels matching the pattern is no less than *θ*|*D*|, where *θ* is a user-specified minimum threshold and |*D*| is the total number of pixels in the data set *D* (We set *θ* = 0.02). Frequent patterns are discovered for each group of recurrence and non-recurrence subjects separately by applying FP-growth^41^. Using frequent patterns, we are now able to deal with any subset of pixels in an efficient and effective way.

### Discriminative patterns

Many of these frequent patterns may not be useful for classification^62^. We obtain discriminative patterns by comparing the frequency of a pattern between two classes (recurring and non-recurring) in two stages: (Figure 2b; see Supplementary Information for details). In the first stage, we quantify the frequency of a pattern for all samples in each class and test if the frequency of a pattern is associated with cancer recurrence. In the second stage, we test if the pattern frequency of the individual subjects in one class (e.g., recurrence) is larger than that in the other class (e.g., non-recurrence). After the two stages, the frequency of the top *m* discriminative patterns (*m* = 100 is set here) in a tissue sample forms an IR feature for the classification algorithm.

### Feature selection

We examine the IR features with respect to the training dataset and select a subset of them to classify the test dataset by adopting a two-stage feature selection scheme. In the first stage, we select a subset of the features that yield a higher relevance with class labels and a lower redundancy among them. In the second stage, the subset is refined by adding new feature(s) and/or removing the already selected feature(s). The detailed description of the feature selection procedure is available in Ref. 45.

### Prediction models

We apply Ranking-Support Vector Machine (SVM)^43^ (Figure 2c) for classification, which assigns relative ranks to any given set of samples. Formally speaking, given two samples, *x_i_* and *x_j_*, the Ranking-SVM computes a function *f* on each sample and assigns ranks *y_i_* and *y_j_* 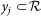 to the two instances by the rule:
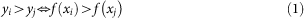
Here, a higher rank indicates greater confidence in the sample being recurrent. Ideally, given a pair of recurrence patient *p*_1_ and non-recurrence patient *p*_2_, the trained SVM should assign a higher rank to any sample of patient *p*_1_ compared to that from patient *p*_2_. The Ranking-SVM algorithm is optimized on the training dataset to minimize the fraction of samples that are mis-ranked.

While the description above formulates a protocol and tests it for a case-control set, translation to a clinical unknown can be incorporated into the same framework by adopting a lookup methodology. We matched the nomogram input data to find the most similar recurrence case and non-recurrence control from the training dataset to an individual patient (query). The similarity between two patients is measured as the inverse of Euclidean distance between their clinical variables – age at prostatectomy, Gleason sum, and pathologic stages (see Supplementary Information for details). Hence, each query returns from the database a recurrent and a non-recurrent match. We rank the query against the two matches via Ranking-SVM. If the query is from a patient who will recur, it will be higher ranked than the non-recurrent sample from the database. If the query is from a non-recurrent patient, it will be ranked lower than the recurrent sample from the database. Quantifying the tendency in the ranks, a preference score for a patient *p*_1_ against a patient *p*_2_ is defined as follows:
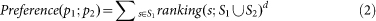
where *S_i_* represents the set of cores samples from a patient *p_i_*, 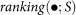 denotes a rank of 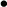 among the set of samples in *S*, and *d* ≥ 1. Since the preference score is dependent on the number of samples of the two patients, a normalized preference score is computed:
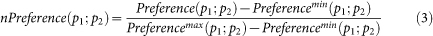
where *Preference^min^*(*p*_1_;*p*_2_) and *Preference^max^*(*p*_1_;*p*_2_) denote the minimum and maximum possible preference scores for the given two patients, respectively. Based on the two preference scores, we predict the probability of recurrence (“IR Score”) of the query patient using a logistic regression model (Figure 2d).

### Kattan nomogram and CAPRA-S score

Kattan nomogram^7^,^8^ is a graphical tool to predict up to 10-year probability of biochemical recurrence after radical prostatectomy. It is constructed based on the Cox proportional hazards regression model. It includes several clinical variables, for instance, PSA level, Gleason grade, lymph node invasion, seminal vesicle invasion, extracapsular extension, surgical margin status, and year of radical prostatectomy. Each variable is represented as a scale, and each scale position corresponds to a prognostic point that shows its prognostic significance in the model. Adding up the prognostic points of all the clinical variables, the total sum is mapped to the probability of biochemical recurrence.

CAPRA-S score^10^ also predicts 5-year biochemical recurrence after radical prostatectomy and uses the Cox proportional hazards regression model. It utilizes PSA level, Gleason score, surgical margin status, extracapsular extension, seminal vesicle invasion, lymph node invasion. Depending on a value of a clinical variable, it assigns a risk point up to 3 for PSA level and Gleason Score, 2 for surgical margin status and seminal vesicle invasion, and 1 for extracapsular extension and lymph node invasion. The minimum and maximum possible score is 0 and 12, respectively. The scores 0–2, 3–5, and ≥6 are considered to be a low-risk, intermediate-risk, and high-risk group, respectively.

### Statistical Analysis

Statistical significance in discriminating pairs of recurrent cases and non-recurrent controls is determined by binomial test with a success probability of 0.5. ROC curves were smoothed prior to computing AUCs to compensate for the underestimation in the empirical AUCs and to estimate the true AUCs^63^. Bootstrap re-sampling with 2000 repetitions is adopted to assess 95% confidence intervals (CI) of AUCs and statistically substantial differences between AUCs of the two ROC curves^63^. Kolmogorov-Smirnov test is employed to examine the differences in score distributions between recurrent cases and non-recurrent controls. Wald chi-square statistic is used to measure the significance of individual predictors in a logistic regression model. The tests were performed using R software version 2.15.2.

## Author Contributions

Conception and design: J.K., A.B., S.S. and R.B. Development of methodology: J.K., S.S. and R.B. Acquisition of data: J.K., A.B., V.M. and M.W. Analysis and interpretation of data: J.K., A.B., V.M., M.W., S.S. and R.B. Writing, review, and/or revision of the manuscript: J.K., A.B., V.M., M.W., S.S. and R.B. Study supervision: S.S. and R.B.

## Supplementary Material

Supplementary Information Supplementary data and methods

## Figures and Tables

**Table 1:** Clinical characteristics of patient cohort in TMAs. The patient characteristics are shown for the calibration dataset of 60 pairs and 82 patients without neoadjuvant or adjuvant therapy and the validation dataset of 66 patients. n and SD denote a number and standard deviation, respectively

		Calibration (n = 120)		Calibration (n = 82)		Validation (n = 66)	
		Cases (n = 60)	Controls (n = 60)	Cases (n = 35)	Controls (n = 47)	Cases (n = 38)	Controls (n = 28)
Age at prostatectomy, mean (SD)		61.6 (7.1)	62.9 (6.5)	62.2 (7.1)	63.1 (6.7)	63.1 (7.1)	63.8 (5.3)
Race, n (%)	White	51 (85)	51 (85)	30 (85.7)	40 (85.1)	33 (86.8)	25 (89.3)
	African American	9 (15)	9 (15)	5 (14.3)	7 (14.9)	4 (10.5)	3 (10.7)
	Other					1 (2.6)	
Gleason sum, n (%)	≤6	12 (20)	12 (20)	7 (20)	10 (21.3)	17 (44.7)	6 (21.4)
	7(3 + 4)	35 (58.3)	35 (58.3)	21 (60)	28 (59.6)	16 (42.1)	15 (53.6)
	7(4 + 3)	7 (11.7)	7 (11.7)	6 (17.1)	6 (12.8)	4 (10.5)	3 (10.7)
	≥8	6 (10)	6 (10)	1 (2.9)	3 (6.4)	1 (2.6)	4 (14.3)
Pathological stage, n (%)	T2a	7 (11.7)	7 (11.7)	4 (11.4)	7 (14.9)	2 (5.3)	2 (7.1)
	T2b	33 (55)	33 (55)	18 (51.4)	30 (63.8)	26 (68.4)	15 (53.6)
	T3a	19 (31.7)	19 (31.7)	13 (37.1)	10 (21.3)	10 (26.3)	11 (39.3)
	T3b	1 (1.7)	1 (1.7)				
Serum PSA ng/mL, mean (SD)		10.6 (8.7)	8.6 (6.0)	10.4 (10.2)	8.7 (6.1)	9.5 (7.2)	8.5 (4.8)
Month to recurrence, mean (SD)		38.6 (29.0)		44.5 (33.1)		36.6 (28.7)	

**Table 2:** Odds ratios for cancer recurrence by quartiles of IR score

Quartile	1 (low)	2	3	4 (high)	P trend
IR OR (95% CI)	1	6.5 (1.3–46.8)	10.8 (2.0–86.8)	21.3 (4.2–168.4)	0.020
CAPRA-S OR (95% CI)	1	3.2 (0.8–15.0)	1.3 (0.3–5.3)	6.1 (1.1–40.3)	0.270
KATTAN OR (95% CI)	1	0.8 (0.2–3.1)	1.5 (0.4–6.5)	1.9 (0.4–10.2)	0.111

OR and CI denote odds ratio and confidence interval, respectively.
