# Supplementary material for: Improving Prediction of Prostate Cancer Recurrence using Chemical Imaging
Source: Sci Rep. 2015 Mar 4;5:8758. doi: 10.1038/srep08758 (PMC4348620; doi:10.1038/srep08758)
Supplement: Supplementary Information — Supplementary data and methods [file srep08758-s1.pdf]

Supplementary Material for

Improving Prediction of Prostate Cancer Recurrence using Chemical Imaging

Jin Tae Kwak[1,2,3], André Kajdacsy-Balla[4], Virgilia Macias[4], Michael Walsh[3,4], Saurabh Sinha[2,*], and Rohit Bhargava[3,5,*]

[1]Center for Interventional Oncology, National Institutes of Health, Bethesda, MD 20892, USA

[2]Department of Computer Science, University of Illinois at Urbana-Champaign, Urbana, IL 61801, USA

[3]Beckman Institute for Advanced Science and Technology, University of Illinois at Urbana-Champaign, Urbana, IL 61801, USA

[4]Department of Pathology, University of Illinois at Chicago, Chicago, IL 60612, USA

[5]Department of Bioengineering, Mechanical Science and Engineering, Electrical and Computer Engineering, Chemical and Biomolecular Engineering and University of Illinois Cancer Center, University of Illinois at Urbana-Champaign, Urbana, IL 61801, USA

*Corresponding Authors:

Saurabh Sinha, PhD, 2122 Siebel Center, 201 N. Goodwin Avenue Urbana, IL 61801, e-mail: sinhas@illinois.edu.

Rohit Bhargava, PhD, 4265 Beckman Institute 405 N. Mathews Avenue Urbana IL 61801, e-mail: rxb@illinois.

Supplementary Methods

Two-stage pattern pruning

A huge number of frequent patterns are, in general, generated by frequent pattern mining methods, but many of these patterns may be indiscriminative or uninformative regarding the given class labels, i.e., recurrence vs. non-recurrence [1]. Examining the entire frequent patterns may not be time- and space-effective. Hence, it is instructive to eliminate the uninformative patterns prior to constructing classification model while retaining the informative ones, called discriminative patterns. To attain the discriminative patterns, we adopt a two-stage pruning method. In the first stage, the frequencies of the patterns between the entire recurrence and non-recurrence subjects are compared via log-odds ratio test. Odds ratio measures how strongly the frequency of a pattern is associated with cancer recurrence. Formally, odds ratio is the ratio of the odds of a pattern present in one group to the odds of its presence in another group. The number of the occurrence of a pattern in the entire recurrence subjects and non-recurrence subjects can be written in the form of a contingency table

	Pattern Exist	Pattern not exist
Recurrence	n_{11}	n_{10}
Non-recurrence	n_{01}	n_{00}

where n_{11} and n_{01} denote the number pixels matching the pattern in the recurrence and non-recurrence subjects, respectively. n_{10} and n_{00} represent the number of pixels which do not own the pattern in the recurrence and non-recurrence subjects, respectively. Then log odds ratio can be computed as

$$L = log\left(\frac{n_{11}n_{00}}{n_{10}n_{01}}\right).$$

Computing the log odds ratio, only the significant patterns (*p-value*<0.01) proceed to the next stage. In the second stage, we compare the frequencies of a pattern among the subjects in recurrence and non-recurrence classes by applying Wilcoxon rank-sum test. It tests if the frequencies of a pattern among the subjects in one group (e.g., recurrence) are larger than those in the other group (e.g., non-recurrence). Ordering the whole subjects by the frequencies of a pattern, a statistic *U* can be computed as counting the number of subjects in one group which are ranked higher than each subject in the other group. The patterns, of which frequencies among the subjects in one group are significantly larger (*p-value*<0.01) than those in the other, are designated as discriminative patterns. The most discriminative top *m* patterns are reported (We set *m*=100).

Search for the most similar patients

In order to predict the outcome of an individual patient (query), we search for the most similar recurrence case and non-recurrence control to the query patient from the training dataset and use them to evaluate the query patient. To find the most similar patients, we compute the similarity between the query patient and each of the entire patients as the inverse of Euclidean distance between clinical variables – age at surgery, Gleason sum, and pathologic stages. Age and Gleason sum are continuous variables. Pathologic stages are considered as discrete variables; for pTNM staging, T2a = 0, T2b = 1, T3a = 2, and T3b = 3; for surgical margin status, extra capsular extension, seminal vesicle involvement, and lymph node involvement, no (or negative) = 0 and yes (or positive) = 1. Prior to computing the similarities, each variable is normalized so that the entire values of the variable range from 0 to 1.

Independence of IR score

The association between IR score and cancer recurrence in consideration of the conventional clinical variables is examined by adopting a logistic regression model. We fit a logistic regression model using IR score and other clinical variables (age at surgery, Gleason grade, pathologic stage, and PSA level) as covariates:

$$\log\frac{P(Y=1)}{P(Y=0)} = \beta_0 + \beta_1 IR + \beta_2 AGE + \beta_3 GRADE + \beta_4 STAGE + \beta_5 PSA$$

where Y is a binary outcome indicating recurrence (1) and non-recurrence (0) and $\beta_0, \dots, \beta_5$ are parameters. IR, AGE, GRADE, STAGE, and PSA denote IR score, age at surgery, Gleason grade, pathologic stage, and PSA level, respectively. Here, $\beta_1, \dots, \beta_5$ estimate conditional odds ratios for the corresponding variables. A conditional odds ratio is odds ratio between a variable and outcome Y as the other variables are held fixed. IR score is added as either continuous or categorical variables. As a continuous variable, the log odds ratio for IR score is estimated as the increase (or change) in the log odds of being recurrence for a one-unit increase in IR score as fixing the other covariates:

$$\log\frac{P(Y=1|IR=k_2, others\ fixed)}{P(Y=0|IR=k_2, others\ fixed)} - \log\frac{P(Y=1|IR=k_1, others\ fixed)}{P(Y=0|IR=k_1, others\ fixed)}$$

where k_1 is an arbitrary value $(0 < x < 1)$ and k_2 is k_1 increased by one-unit in IR score. As a categorical variable, patients are assigned to quartiles (1-4) by IR score; the higher IR score, the larger quartile is assigned. Fixing the other covariates, the odds ratio is computed for each of the three quartiles (2-4) compared to the lowest quartile (1); for example, the odds ratio for the highest quartile is estimated as the ratio of the odds of being recurrence for that quartile to the odds of being recurrence for the lowest quartile:

$$\frac{P(Y=1|IR=4, others\ fixed)/P(Y=0|IR=4, others\ fixed)}{P(Y=1|IR=1, others\ fixed)/P(Y=0|IR=1, others\ fixed)}.$$

Supplementary Figures

Figure S1.

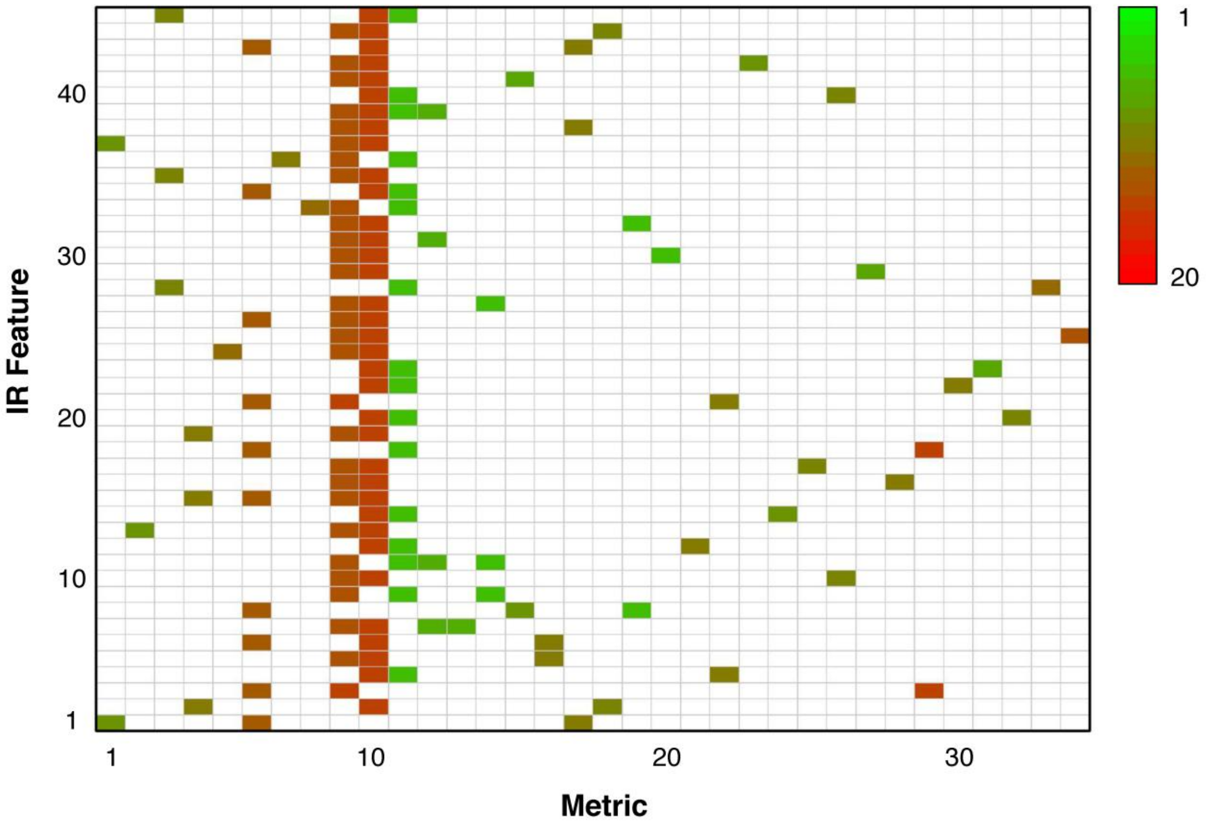

Figure S1. IR feature map. Metrics associated with the IR stroma features are shown. Each row represents an IR feature. Each column denotes a metric. 34 metrics are ordered as appeared in Table S2. Bins are marked with different colors.

Supplementary Tables

Table S1. Odds ratios for cancer recurrence by quartiles of IR score.

Quartile	1 (low)	2	3	4 (high)	P trend
IR OR (95% CI)	1	6.485 (1.315-46.838)	10.863 (2.022-86.781)	21.307 (4.150-168.446)	0.020
CAPRA-S OR (95% CI)	1	3.248 (0.758-15.041)	1.260 (0.282-5.318)	6.132 (1.116-40.342)	0.270
KATTAN OR (95% CI)	1	0.811 (0.198-3.100)	1.509 (0.350-6.514)	1.928 (0.379-10.186)	0.111

OR and CI denote odds ratio and confidence interval, respectively.

Table S2. Description of metrics.

Metric Type	Numerator Position or Region (cm^{-1})	Denominator Position or Region (cm^{-1})	IR Feature (Yes/No)	Assignment
Absorbance Ratio	3088	1400	Yes	O-H stretching [2],
Absorbance Ratio	3088	1450	Yes	N-H stretching,
Absorbance Ratio	3288	1400	Yes	Protein [3]
Absorbance Ratio	3288	1544	Yes	
Absorbance Ratio	3288	1652	Yes	
Peak-to-Area Ratio	3288	1504–1586	Yes	
Area-to-Peak Ratio	3030–3600	1544	Yes	
Area-to-Area Ratio	3030–3600	1592–1738	Yes	

Center of Gravity	3200–3400		Yes	
Center of Gravity	3030–3600		Yes	
Absorbance Ratio	1042	1450	Yes	C-O stretching,
Absorbance Ratio	1042	1544	Yes	Oligosaccharide [4]
Absorbance Ratio	1042	1652	Yes	
Area-to-Peak Ratio	992–1110	1544	Yes	
Center of Gravity	998–1052		Yes	
Center of Gravity	998–1132		Yes	
Absorbance Ratio	966	1544	Yes	C-O stretching,
Peak-to-Area Ratio	966	1504–1586	Yes	Nucleic acid [5]
Absorbance Ratio	1080	1450	Yes	Symmetry phosphate stretching,
Absorbance Ratio	1080	1652	Yes	Nucleic acid [6], Glycogen [7]
Peak-to-Area Ratio	1170	1504–1586	Yes	Nucleic acid [8]
Area-to-Peak Ratio	1150–1176	1544	Yes	
Absorbance Ratio	1336	1080	Yes	CH_2 wagging [9],
Absorbance Ratio	1334	1652	Yes	Collagen [10]
Peak-to-Area Ratio	1336	1504–1586	Yes	
Area-to-Peak Ratio	1326–1348	1544	Yes	
Absorbance Ratio	1388	1652	Yes	COO- symmetric stretching,
Peak-to-Area Ratio	1390	1504–1586	Yes	fatty acids and amino acids [11]
Center of Gravity	1366–1426		Yes	
Absorbance Ratio	1462	1400	Yes	CH_2 scissoring [12], CH2 bending,
Absorbance Ratio	1452	1652	Yes	amino acids [13]
Area-to-Peak Ratio	1424–1474	1544	Yes	
Peak-to-Area Ratio	1564	1504–1586	Yes	Amide II [14]

Peak-to-Area Ratio	1652	1504–1586	Yes	Amide I [15]
Absorbance Ratio	3430	1080	No	O-H stretching [2],
Absorbance Ratio	3288	1080	No	N-H stretching,
Absorbance Ratio	3208	1080	No	Protein [3]
Absorbance Ratio	3288	1400	No	
Absorbance Ratio	3088	1544	No	
Absorbance Ratio	3088	1652	No	
Peak-to-Area Ratio	3088	1504–1586	No	
Area-to-Peak Ratio	3030–3140	1544	No	
Absorbance Ratio	1080	1400	No	Symmetry phosphate stretching,
Absorbance Ratio	1080	1544	No	Nucleic acid [6], Glycogen [7]
Absorbance Ratio	936	1544	No	
Peak-to-Area Ratio	936	1544–1586	No	
Absorbance Ratio	1170	1080	No	Nucleic acid [8]
Absorbance Ratio	1336	1544	No	CH_2 wagging [9],
Area-to-Area Ratio	1326–1348	1592–1738	No	Collagen [10]
Absorbance Ratio	1388	1080	No	COO- symmetric stretching,
Absorbance Ratio	1390	1544	No	fatty acids and amino acids [11]
Area-to-Peak Ratio	1372–1422	1544	No	
Area-to-Area Ratio	1372–1422	1592–1738	No	
Absorbance Ratio	1462	1450	No	Methylene deformation [16], CH_2
Absorbance Ratio	1462	1544	No	scissoring [12], CH2 bending,
Absorbance Ratio	1452	1080	No	amino acids [13]
Absorbance Ratio	1450	1544	No	
Peak-to-Area Ratio	1450	1504–1586	No	

Peak-to-Area Ratio	1462	1504–1586	No	
Area-to-Area Ratio	1424–1474	1592–1738	No	
Center of Gravity	1426–1470		No	
Absorbance Ratio	1564	1544	No	Amide II [14 17]
Absorbance Ratio	1562	1652	No	
Absorbance Ratio	1536	1652	No	
Peak-to-Area Ratio	1536	1592–1738	No	
Peak-to-Area Ratio	1544	1592–1738	No	
Area-to-Peak Ratio	1504–1586	1544	No	
Center of Gravity	1504–1584		No	
Absorbance Ratio	1656		No	Amide I [15 18 14]
Absorbance Ratio	1648		No	
Absorbance Ratio	1632		No	
Absorbance Ratio	1630		No	
Area-to-Peak Ratio	1592–1738	1544	No	
Center of Gravity	1592–1738		No	
Absorbance Ratio	1720	1652	No	
Absorbance Ratio	1718	1544	No	
Absorbance Ratio	1402	1080	No	Asymmetric CH3 bending of methyl groups of protein[19], C-N stretching, N-H deformation, C-H deformation[20]
Absorbance Ratio	1402	1652	No	
Absorbance Ratio	1400	1544	No	COO- group, fatty acids and amino acids [16].
Peak-to-Area Ratio	1400	1504–1586	No	
Absorbance Ratio	1312	1080	No	Amide III band components of

Absorbance Ratio	1312	1544	No	proteins[19]
Peak-to-Area Ratio	1312	1504–1586	No	
Area-to-Peak Ratio	1302–1326	1544	No	
Area-to-Area Ratio	1302–1326	1592–1738	No	
Absorbance Ratio	1280	1080	No	Amide III[21], collagen[6]
Absorbance Ratio	1280	1544	No	
Peak-to-Area Ratio	1280	1504–1586	No	
Absorbance Ratio	1236	1544	No	Amide III, asymmetric
Peak-to-Area Ratio	1236	1504–1586	No	phosphodiester stretching,
				mainly from nucleic acids[18]
Area-to-Peak Ratio	1206–1290	1544	No	Amide III[7 21], collagen[22 8]
Area-to-Area Ratio	1206–1290	1592–1586	No	
Center of Gravity	1194–1218		No	Amide III[21], collagen[22]
Center of Gravity	1194–1286		No	
Absorbance Ratio	1160	1544	No	C-O stretching[23]
Absorbance Ratio	1120	1080	No	?(1000–1150 cm^{-1} nucleic acids[21])
Absorbance Ratio	1062	1544	No	Ribose/deoxyribose C-O
Center of Gravity	1050–1100		No	stretching[24]

Metric definitions and assignments of the numerator bands are provided. IR Feature column shows 34 metrics which were selected and used to generated IR stromal features.

Supplementary References

1 Hong, C., Xifeng, Y., Jiawei, H., Chih-Wei, H. Discriminative Frequent Pattern Analysis for Effective Classification. *Proc ICDE 2007*, Istanbul, Turkey, 716-725, IEEE (doi: 10.1109/icde.2007/367917).

2 Chalmers, J. M., Griffiths, P. R. *Handbook of vibrational spectroscopy*. (J. Wiley, New York, 2002).

3 Skornyakov, I. V., Tolstorozhev, G. B., Butra, V. A. Infrared Absorption Spectra of Human Malignant Tumor Tissues. *Journal of Applied Spectroscopy* **75**, 420-425 (2008).

4 Severcan, F., Kaptan, N., Turan, B. Fourier transform infrared spectroscopic studies of diabetic rat heart crude membranes. *Spectroscopy-an International Journal* **17**, 569-577 (2003).

5 Dovbeshko, G. I., Gridina, N. Y., Kruglova, E. B., Pashchuk, O. P. FTIR spectroscopy studies of nucleic acid damage. *Talanta* **53**, 233-246 (2000).

6 Benedetti, E. *et al.* Infrared Characterization of Nuclei Isolated from Normal and Leukemic (B-Cll) Lymphocytes .3. *Applied spectroscopy* **40**, 39-43 (1986).

7 Wang, H. P., Wang, H. C., Huang, Y. J. Microscopic FTIR studies of lung cancer cells in pleural fluid. *Science of the Total Environment* **204**, 283-287 (1997).

8 Holman, H.-Y. N. Detecting exposure to environmental organic toxins in individual cells: toward development of a microfabricated device. **3606**, 55-62, doi:10.1117/12.350062 (1999).

9 Mordechai, S. Fourier-transform infrared spectroscopy of human cancerous and normal intestine. **3918**, 66-77, doi:10.1117/12.384956 (2000).

10 Rieppo, L. *et al.* Application of second derivative spectroscopy for increasing molecular specificity of fourier transform infrared spectroscopic imaging of articular cartilage. *Osteoarthritis and Cartilage* **20**, 451-459, doi:DOI 10.1016/j.joca.2012.01.010 (2012).

11 Palaniappan, P. L. R. M., Vijayasundaram, V. FTIR Study of Arsenic Induced Biochemical Changes on the liver Tissues of Fresh Water Fingerlings *Labeo rohita*. *Rom. J. Biophys.* **18**, 135-144 (2008).

12 O Faolain, E. *et al.* The potential of vibrational spectroscopy in the early detection of cervical cancer: an exciting emerging field. **5826**, 25-36, doi:10.1117/12.603344 (2005).

13 Scott, D. A. *et al.* Diabetes-related molecular signatures in infrared spectra of human saliva. *Diabetology & Metabolic Syndrome* **2** (2010).

14 Yang, D. *et al.* A Fourier-Transform Infrared Spectroscopic Comparison of Cultured Human Fibroblast and Fibrosarcoma Cells: A New Method for Detection of Malignancies. *J. Clin. Laser Med. Surg.* **13**, 55-59 (1995).

15 Diem, M., Boydston-White, S. & Chiriboga, L. Infrared spectroscopy of cells and tissues: Shining light onto a novel subject. *Applied spectroscopy* **53**, 148a-161a (1999).

16 Wood, B. R., Quinn, M. A., Burden, F. R. & McNaughton, D. An investigation into FTIR spectroscopy as a biodiagnostic tool for cervical cancer. *Biospectroscopy* **2**, 143-153 (1996).

17 Huleihel, M. *et al.* Novel spectral method for the study of viral carcinogenesis in vitro. *Journal of Biochemical and Biophysical Methods* **50**, 111-121, doi:10.1016/s0165-022x(01)00177-4 (2002).

18 Khamis, Z. I., Sahab, Z. J., Byers, S. W., Sang, Q. X. Novel stromal biomarkers in human breast cancer tissues provide evidence for the more malignant phenotype of estrogen receptor-negative tumors. *J Biomed Biotechnol* **2011**, 723650, doi:10.1155/2011/723650 (2011).

19 Eberlin, L. S. *et al.* Molecular assessment of surgical-resection margins of gastric cancer by mass-spectrometric imaging. *Proc Natl Acad Sci U S A* **111**, 2436-2441, doi:10.1073/pnas.1400274111 (2014).

20 Kong, K. *et al.* Diagnosis of tumors during tissue-conserving surgery with integrated autofluorescence and Raman scattering microscopy. *Proc Natl Acad Sci U S A* **110**, 15189-15194, doi:10.1073/pnas.1311289110 (2013).

21 Freudiger, C. W. *et al.* Label-free biomedical imaging with high sensitivity by stimulated Raman scattering microscopy. *Science* **322**, 1857-1861, doi:10.1126/science.1165758 (2008).
22 Ricciardelli, C. *et al.* Elevated stromal chondroitin sulfate glycosaminoglycan predicts progression in early-stage prostate cancer. *Clinical cancer research : an official journal of the American Association for Cancer Research* **3**, 983-992 (1997).
23 Punnen, S. *et al.* Multi-institutional validation of the CAPRA-S score to predict disease recurrence and mortality after radical prostatectomy. *Eur Urol* **65**, 1171-1177, doi:10.1016/j.eururo.2013.03.058 (2014).
24 Bianco, F. J., Jr. *et al.* Radical prostatectomy nomograms in black American men: accuracy and applicability. *J Urol* **170**, 73-76; discussion 76-77, doi:10.1097/01.ju.0000068037.57553.54 (2003).
